# An improved BECT spike detection method with functional brain network features based on PLV

**DOI:** 10.3389/fnins.2023.1150668

**Published:** 2023-03-16

**Authors:** Lurong Jiang, Qikai Fan, Juntao Ren, Fang Dong, Tiejia Jiang, Junbiao Liu

**Affiliations:** ^1^School of Information Science and Engineering, Zhejiang Sci-Tech University, Hangzhou, China; ^2^College of Information and Electric Engineering, Zhejiang University City College, Hangzhou, China; ^3^Department of Neurology, The Children's Hospital, Zhejiang University School of Medicine, National Clinical Research Center for Child Health, Hangzhou, China; ^4^Digital Culture Innovation Research Institute, Zhejiang University City College, Hangzhou, China

**Keywords:** spike detection, functional brain networks, phase locking value, network structure features, ANN

## Abstract

**Background:**

Children with benign childhood epilepsy with centro-temporal spikes (BECT) have spikes, sharps, and composite waves on their electroencephalogram (EEG). It is necessary to detect spikes to diagnose BECT clinically. The template matching method can identify spikes effectively. However, due to the individual specificity, finding representative templates to detect spikes in actual applications is often challenging.

**Purpose:**

This paper proposes a spike detection method using functional brain networks based on phase locking value (FBN-PLV) and deep learning.

**Methods:**

To obtain high detection effect, this method uses a specific template matching method and the ‘peak-to-peak' phenomenon of montages to obtain a set of candidate spikes. With the set of candidate spikes, functional brain networks (FBN) are constructed based on phase locking value (PLV) to extract the features of the network structure during spike discharge with phase synchronization. Finally, the time domain features of the candidate spikes and the structural features of the FBN-PLV are input into the artificial neural network (ANN) to identify the spikes.

**Results:**

Based on FBN-PLV and ANN, the EEG data sets of four BECT cases from the Children's Hospital, Zhejiang University School of Medicine are tested with the AC of 97.6%, SE of 98.3%, and SP 96.8%.

## 1. Introduction

Epilepsy is a common neurological disease caused by temporary brain dysfunction resulting from the abnormal discharge of brain cells. Benign childhood epilepsy with centro-temporal spikes (BECT) is one of the most common epilepsy syndromes in children, accounting for about 23% of childhood epilepsy (Shi et al., 2020). The onset age of BECT patients is usually about 3–14 years, and the disease will slowly subside in their teens. Usually, the seizures of BECT patients are considered benign. However, according to recent studies, compared with healthy children of the same age and gender, the problems of attention deficit and hyperactivity disorder in BECT patients are more significant (Tovia et al., 2011). The common characteristics of the patients are seizures in the focal sensorimotor function area in childhood, and a large number of spike discharges near the central and centro-temporal regions during the attack period (Kirby et al., 2017). The diagnosis of epileptic diseases is mainly through clinical history and electroencephalogram (EEG) examination. Through further data analysis and clinical practice, it has been found that the location and duration ratio of Rolandic region epileptiform discharges in the entire EEG monitoring have gradually become the main indicators for diagnosing patients with BECT syndrome and determining the need for medication (Xu et al., 2021). This indicator has become an important criterion for determining whether BECT patients require drug therapy. Therefore, detecting spikes can help doctors make more accurate diagnoses and treatments for BECT patients. In hospitals, EEG technicians usually read pictures and identify spikes by manual recognition. However, the detection results are often different due to the subjective factors of the inspectors. With the increased number of EEG records, there will inevitably be misjudgments caused by visual fatigue and other factors. Therefore, design and apply the automatic detection algorithm of epileptic spikes can effectively improve the accuracy and efficiency of diagnosis (Benbadis et al., 2020).

In recent years, with the rapid development of signal analysis and computer-aided intelligent diagnosis technology, research in the direction of spikes detection has also made progress. Gloor (1975) morphologically defined spikes in EEG as : (a) A triangular transient with an amplitude at least twice the background signal of the first 5 s of any channel in the EEG signal. (b) At least 200 *ms* duration. (c) Including the presence of a field defined by the participation of the second adjacent electrode. Tzallas et al. (2006) used the Kalman filter to estimate the autoregressive parameters according to the non-stationarity of EEG signals, established a spike detection model, and set the detection sensitivity threshold. The filter output signal higher than the threshold is regarded as the spike position, so this method is difficult to avoid the problem of false positive spikes (FPS). Nonclercq et al. (2012) presented an automatic detection algorithm based on time K-Means clustering, which clusters candidate spikes, extracts the centroid of the cluster to generate a new matching template. Jiang et al. (2021) proposed an improved spike detection algorithm based on multiple template matching and feature extraction. First, candidate spikes set is obtained through a general morphological template, time domain features are extracted, and candidate spikes are filtered by a threshold method. Finally, the K-Means method obtains a specific template for spike detection. Wu et al. (2022) proposed a spike detection algorithm based on optimal template and morphological feature, which optimized the parameters of template according to the EEG data of patients and used morphological features to reduce FPS. However, due to the large difference in EEG signals of different patients in time and space, the number of template libraries is large in actual detection, and the detection of epileptic spikes in different patients cannot be flexibly realized.

Since the nineteenth century, people have realized that the brain has a complex network structure (Lynn and Bassett, 2019). The brain needs information interaction in multiple brain regions when completing body movements, and different functional regions are interconnected to form a brain network. Different studies differ in the selection of EEG signal types and the way connections are established, but all have one thing in common, using tools in the field of complex networks for analysis (Leitgeb et al., 2020). Graph theory is a mathematical way to analyze complex networks quantitatively. Complex networks can be described as graphs *G*(*N, K*) composed of *N* nodes and *K* connections or edges. The specificity of brain networks can be found by measuring the multi-functional scale of the graph *G* (Song et al., 2015). Netoff et al. (2004) successfully explained the etiology of epilepsy using functional brain networks, and the complex network method was valued in epilepsy research. The brain is a very complex network system, with high-intensity connections between functional areas and different small-world networks between different functional areas (Nemzer et al., 2021). For the functional network established by EEG, we use the knowledge of graph theory to analyze the topological characters of the functional brain network by using complex network measures, such as degree, clustering coefficient, and global efficiency. In recent years, more and more studies have shown that seizures affect the local and global characteristics of human brain activity. Frassineti et al. (2021) constructed a complex network by EEG data of neonatal epilepsy patients. They explored the changes in brain properties during seizures by studying the Synchronizability (S) index and the functional brain network's Circular Omega Complexity (COC). Ahmadi et al. (2020) used short-term EEG data to detect seizures by analyzing features such as signals, functional brain networks, and EEG microstate features. Jiang et al. (2023) constructed the PCC and MI combined functional brain networks (PMNet) based on the Pearson coefficient (PCC) and mutual information (MI), used the complex network to calculate brain network features, and detected epileptic seizures.

As a new research direction of machine learning, deep learning has significant advantages in effectiveness and practicability. It has also made a series of progress in seizure detection in recent years. Wang et al. (2021) designed a convolutional neural network (CNN) with three-dimensional kernels, which achieved good results on CHB-MIT. Abdelhameed et al. (2018) proposed an automatic epilepsy detection system that uses a one-dimensional CNN as a preprocessing front-end and a Bi-directional long short-term memory (Bi-LSTM) as a preprocessing back-end. The system can effectively classify EEG signals. Hu et al. (2019) divided the amplitude spectrum of multi-channel EEG signals into 19 frequency sub-band features, used the CNN algorithm to extract features, and combined them with the support vector machine (SVM) for classification. Based on geometric deep learning (GDL), Dissanayake et al. (2021) extracted networks from EEG data for epilepsy prediction. Usman et al. (2021) automatically extracted seizure characteristics through a three-layer CNN and used a LSTM for seizure prediction. The typical characteristics of epileptic seizures are the abnormal firing of neurons and synchronous firing of action potentials, which are presented as epileptic spikes in neuroelectric signals. For patients with BECT and other types of epilepsy, the localization analysis of epileptic spikes is more meaningful than seizure detection. In order to better diagnose patients, neurologists need to analyze a large number of EEG data to find millisecond-level epileptic spike discharges, which is extremely cumbersome and time-consuming (Yan et al., 2018). Therefore, positioning monitoring and recording analysis of various spike signals for the location of epileptic regions, the precise mechanism of epilepsy, and even predict of seizures are important (Chahid et al., 2020). Fukumori et al. (2019) inputted the original EEG signal into the convolutional layer to extract the feature frequency band. Then, the CNN and the recurrent neural network (RNN) models were used to detect epileptic spikes. Prasanth et al. (2020) inputted δ, θ, α, β, and full frequency bands into a CNN to detect epileptic spikes. Abou Jaoude et al. (2020) trained a CNN to identify medial temporal lobe (MTL) epileptic spikes in a single intracranial bipolar channel, with great potential for accurate detection and localization of MTL epileptic spikes. Xu et al. (2021) proposed a BECT epileptic spike detection algorithm with sequence features of EEG and LSTM classifier, which has high detection sensitivity.

This paper proposes an improved BECT spike intelligent detection method using phase locking value (PLV). The main works of this paper are: (a) According to the morphological characteristics of spikes, this method is used to establish the functional brain network based on PLV (FBN-PLV), and the spike characters of the functional brain network perspective are extracted. (b) Then, a spike detection method is proposed based on functional brain network features and deep learning. The time domain constructed by spikes morphology and functional brain network features are extracted and classified by artificial neural network (ANN). The proposed method has achieved high sensitivity in actual BECT patient data, confirming that the method has great clinical value.

The structure of this paper is as follows: The second section proposes the overall algorithm framework of this paper and introduces the extraction of temporal features of spikes, the construction of functional brain networks, and the analysis of network topology attributes. In the third section, the ANN model is used to test the performance of the whole algorithm, and the experimental results are analyzed. The fourth section compares this algorithm with other algorithms. The fifth section summarizes this paper and looks forward to future work.

## 2. Materials and methods

### 2.1. Framework of the method

**Figure 1:**
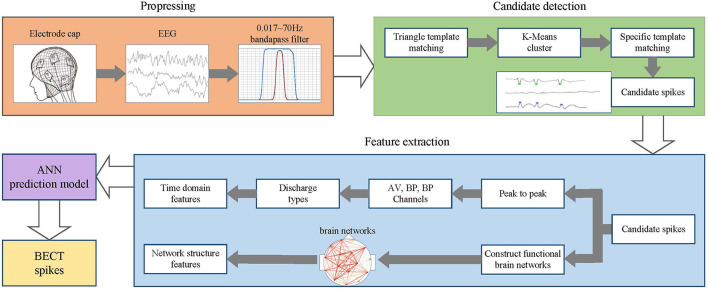
Flowchart of BECT spike detection algorithm based on FBN-PLV.

The overall process of this method is shown in Figure 1. In the preprocessing stage, the EEG data of BECT patients are collected, and a 0.017~70 *HZ* band-pass filter processes the EEG signal. In the candidate spike detection stage, the triangle template matching method filters the EEG signal. When the cross-correlation and morphological features of the signal frame exceed the threshold, the spike sequence is extracted, and the candidate spike is obtained by K-Means clustering and specific template matching. In the feature extraction stage, the time domain features and spike functional brain network features are extracted from the candidate spikes. Finally, these features are used as the input of ANN for detecting and recognizing spikes.

### 2.2. Candidate spike detection with triangle template matching

In EEG mapping, spikes have an apparent geometric meaning, so they can be screened by template matching to obtain a candidate spike set (Gloor, 1975). The specific steps are as follows :

(1) Triangle template matching

First, reset the spike template based on the triangle template for waveform screening. Then calculate the mutual correlation between a given template and all signal frames. The calculation method is shown in Equation (1):


(1) $$\begin{array}{l} C=\frac{\overset{N}{\underset{n=1}{\sum }}y\left(n\right)\times f\left(n\right)}{\sqrt{\overset{N}{\underset{n=1}{\sum }}y^{2}\left(n\right)}\sqrt{\overset{N}{\underset{n=1}{\sum }}f^{2}\left(n\right)}} \end{array}$$

where *y*(*n*) is the template, *f*(*n*) is the signal to be measured, and *N* is the length of EEG. The universal template is a spike template composed of a triangle with an amplitude of 300 μ*V* and a duration of 60 *ms*, and the calculated window width is 300 *ms*. Finally, extract the morphological features with rising slope, falling slope, and curvature of the spike. The cross-correlation threshold is set to 60%, and the morphological feature threshold is set to 30%. If both the correlation and the feature threshold exceed the set value, this EEG signal frame is considered a spike sequence (Chatrian, 1974).

(2) K-Means clustering

The peak sequence obtained by triangle template matching is classified into clusters by the K-Means clustering method. The number of clusters is automatically determined. Starting from a cluster, the number of clusters increases until the number of candidate spikes in the cluster is less than 5% of the total number of spikes. If the number of candidate spikes in a cluster is less than 5% of the total number of spikes, the cluster will be discarded, and the remaining clusters constitute the final result of K-Means clustering.

(3) Specific template matching

The centroid of the selected K-Means cluster is used as a specific template, and a new correlation and feature threshold is set. The template matching is performed again to obtain different types of candidate spikes. The matching results of all specific templates are added as the final candidate spike set. Since various centroids can detect a single spike, an interval threshold is set. If the interval between two neighbor spikes is less than this threshold, the two spikes are considered as one spike.

### 2.3. Morphological time domain characteristics of spike

Montage is the arrangement channels on the EEG machine display, defined by the exploring and reference electrodes. The two most common types of montages in EEG signals are bipolar montages and referential montages. The Montage indicates that different lead combinations can record EEG signals from different amplifiers. The reference montage and the bipolar Montage correspond to the reference lead method and the bipolar lead method, respectively. All recording electrodes of the reference lead method are connected to the negative end of the amplifier, and the reference electrode is connected to the positive end. Most of the earlobe are not in direct contact with the scalp surface. The potential is relatively weak. It is often used as EEG reference electrode. The bipolar lead method is two recording electrodes connected to the two ends of the preamplifier. EEG forms the potential difference between the two, thereby amplifying the local potential, making it easier to eliminate external noise (Koubeissi and Azar, 2017).

**Figure 2:**
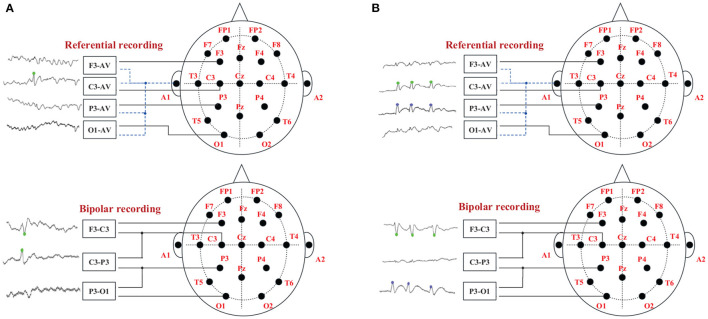
The “peak-to-peak” phenomenon of different discharge modes. The green dots on the EEG signals indicate spike discharge in the C3 channel, and the blue dots indicate spike discharge in the P3 channel. **(A)** Single channel discharge. **(B)** Multiple channel discharge.

There are usually two ways of spike discharge in two average (AV) channels in patients' EEG signals, and their relationship with two adjacent bipolar (BP) channels is shown in Figure 2. Figure 2A represents a single channel discharge, there is only one channel spike discharge, and only spikes are detected on C3-AV. The two related BP channels are F3-C3 and C3-P3; Figure 2B represents multi-channel discharge, both channels have spike discharge, and spikes are detected on both C3-AV and P3-AV. The two related BP channels are F3-C3 and C3-P3. Both of the above two spike discharge modes show a “peak-to-peak” phenomenon (Wang et al., 2020).

**Figure 3:**
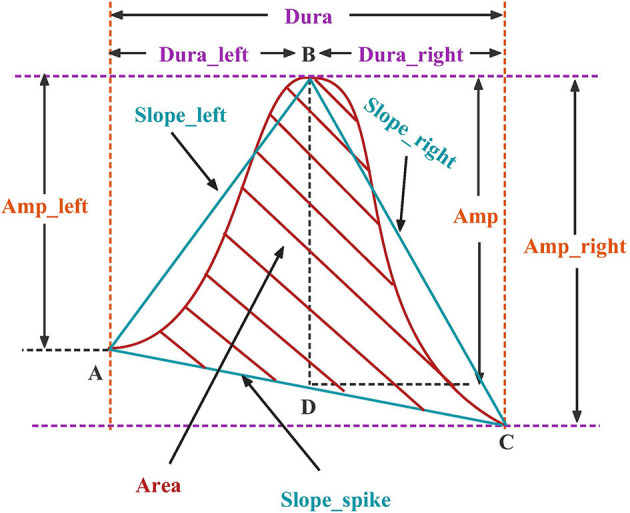
Candidate spike time domain feature.

Each candidate spike obtained by template matching can determine an AV channel and two BP channels by the 'peak-to-peak' phenomenon, and then extract the time-domain features of these three channels. The peak is divided into the left, right half, and whole waves. The time domain features are divided into four categories, as shown in Figure 3, including duration, amplitude, slope and area. Each channel has 10 spikes features and three channels have a total of 3 × 10 features, as listed in Table 1.

**Table 1:** Size of candidate spike morphological characteristics and FBN-PLV feature.

**Type**	**Feature**	**Size**
Time domain feature	Dura_left, Dura_right, Dura_peaks	3 × 3
	Amp_left, Amp_right, Amp_peaks	3 × 3
	Slope_left, Slope_right, Slope_peaks	3 × 3
	Area_peaks	1 × 3
Network structure feature	AD, ACC, CPL, GE, MD	5 × 1

### 2.4. Functional brain network based on PLV

#### 2.4.1. PLV

Phase synchronization exists widely in EEG signals, and PLV is one of the common methods to measure phase synchronization. It represents the absolute value of the average phase difference between any two signals, which can be used to reflect the instantaneous phase relationship between two signals. The PLV indicates the synchronization of the two signals and is used to construct functional brain networks (Ding et al., 2022). Compared to PCC, MI, and PLI, the FBN-PLV is more appropriate for non-stationary and nonlinear signals such as EEG. The brain is a nonlinear and dynamic system, so it is reasonable to use the phase-locked loop to measure the internal activity of the brain since it is robust to amplitude fluctuations. The FBN-PLV can differentiate the phase component from the amplitude component, making it more suitable for EEG, which is susceptible to muscle activity and blinks (LeCun et al., 1998).

The Hilbert transform is often used to calculate the instantaneous value of the signal, which decomposes the amplitude and phase. *H*(*x*(*t*)) is defined as the Hilbert transform of the filtered electrode signal *x*(*t*), as shown in Equation (2). Then *H*(*x*(*t*)) is decomposed, as shown in Equation (3), where $\Phi_{x}^{H}\left(t\right)$ is the phase of the signal *x*(*t*), which is defined in Equation (4). Similarly, the phase $\Phi_{y}^{H}\left(t\right)$ of signal y(t) can be obtained by the Hilbert transform of signal *y*(*t*). If the phase locking ratio of signal *x*(*t*) and *y*(*t*) is *p*:*q*, then the phase difference between the two signals is defined as shown in Equation (5). In this paper, the contribution of the two signals is equal, so *p* and *q* are set to 1.


(2) $$\begin{array}{l} H\left(x\left(t\right)\right)=\frac{1}{\pi }\int_{-\infty }^{+\infty }\frac{x\left(t^{\prime }\right)}{t-t^{\prime }}\text{d}t^{\prime } \end{array}$$

(3) $$\begin{array}{l} Z_{x}\left(t\right)=x\left(t\right)+iH\left(x\left(t\right)\right)=A_{x}^{H}\left(t\right)\exp\left(i\Phi_{x}^{H}\left(t\right)\right) \end{array}$$

(4) $$\begin{array}{l} \Phi_{x}^{H}\left(t\right)=\arctan\left(\frac{Im\left\{Z_{x}\left(t\right)\right\}}{Re\left\{Z_{x}\left(t\right)\right\}}\right)=\arctan\left(\frac{H\left(x\left(t\right)\right)}{x\left(t\right)}\right) \end{array}$$

(5) $$\begin{array}{l} \Phi_{xy}^{H}\left(t\right)=p\Phi_{x}^{H}\left(t\right)-q\Phi_{y}^{H}\left(t\right) \end{array}$$

Assuming that the total number of sample points in this time period is *N*, $\Phi_{xy}^{H}\left(t,n\right)$ represents the instantaneous phase difference between *x*(*t*) and *y*(*t*) at point *n*, the PLV for two continuous signals *x*(*t*) and *y*(*t*) at time *t* is defined as:


(6) $$\begin{array}{l} PLV\left(t\right)=\frac{1}{N}|\overset{N}{\underset{n=1}{\sum }}\exp\left(i\left(\Phi_{xy}^{H}\left(t,n\right)\right)\right)| \end{array}$$

#### 2.4.2. Construct functional brain networks based on PLV

**Figure 4:**
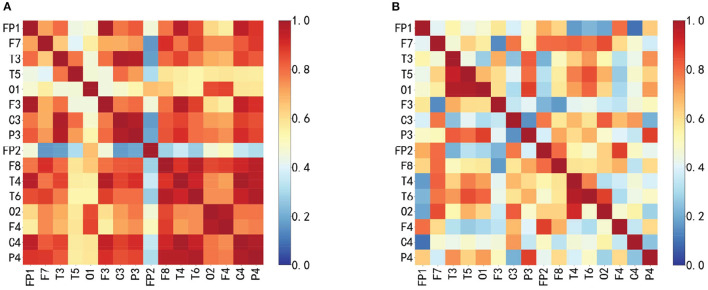
Functional brain network association matrix, horizontal axis and vertical axis represent EEG lead combination, the element value in the matrix is the PLV between any two node signals, the PLV range is [0, 1], blue represents the PLV is small, red represents the PLV is large. **(A)** Spike discharge. **(B)** Nospike discharge.

**Figure 5:**
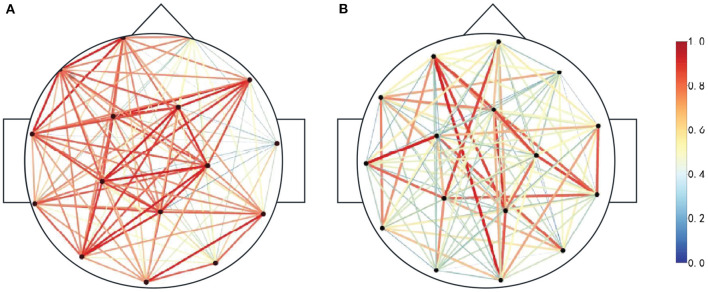
Functional brain network connections. The darker the color represents the weight of each side, the more red connections indicate the stronger signal phase synchronization between the two nodes, and the more bluer connections indicate the weaker signal phase synchronization between the two nodes. **(A)** Spike discharge. **(B)** Nospike discharge.

Since the duration of the spikes is generally 20~70 *ms*, the frequency is 14.5~70 *Hz*, and if the PLV time window is too large to ensure signal stability, the sliding time window of 100 *ms* and the step length of 100 *ms* are selected to calculate PLV. In this paper, scalp EEG channels are selected as nodes. The functional brain network is constructed according to the absolute value of the PLV correlation index between any two channels in the candidate spike segments, and the phase synchronization functional network matrix is obtained, as shown in Figure 4. When PLV is 0, there is no phase synchronization between the two signals. When PLV is 1, the two signals are completely synchronized. Figure 4A is the functional brain network when the actual spike discharge in the candidate spikes, and Figure 4B is the functional brain network when the FPS discharge in the candidate spikes. Compared Figures 4A, B, it can be concluded that the cerebral cortex has strong phase synchronization and high information interaction efficiency during spikes discharge. Figure 5A is the brain network connection when the actual spike discharge in the candidate spikes, and Figure 5B is the brain network connection when the FPS discharge in the candidate spikes. The results show that the network connection is stronger, and the information is more prosperous when the spike is discharged.

#### 2.4.3. Features of FBN-PLV

After constructing the FBN-PLV, the connection weights are retained. The weighted complex brain network topology attributes are analyzed by the graph theory method, including the average degree (AD), the average clustering coefficient (ACC), the characteristic path length (CPL), the modularity (MD), and the global efficiency (GE), as shown in Table 1.

Degree is graph theory's most basic index of statistical complex network topology attributes. The degree of a node represents the number of edges connected to other nodes and the sum of information transmission of the node. The larger the degree value, the more influential the node is in the network structure. In the weighted network, *a*_*ij*_ represents the connection between node *i* and node *j*, and *D*_*i*_ represents the degree of node *i*, as shown in Equation (7). The AD in the network is defined as the average value of all *N* node degrees. As shown in Equation (8), it can represent the network's sparsity. The larger the average degree value, the more edges there are between nodes in the network, and the denser the street edges, and vice versa.


(7) $$\begin{array}{l} D_{i}=\overset{N}{\underset{j-1}{\sum }}a_{ij} \end{array}$$

(8) $$\begin{array}{l} AD=\frac{1}{N}\overset{N}{\underset{i}{\sum }}D_{i} \end{array}$$

Clustering coefficient measures the degree of aggregation of nodes in the network, which can reflect the density between neighbor nodes of a network node. In a network, the larger the clustering coefficient of a node is, the more influential the node is in the network. The definition of the clustering coefficient is shown in Equation (9).


(9) $$\begin{array}{l} C_{i}=\frac{2|e_{i}|}{k_{i}\left(k_{i}-1\right)} \end{array}$$

(10) $$\begin{array}{l} ACC=\frac{1}{N}\overset{N}{\underset{i}{\sum }}C_{i} \end{array}$$

where |*e*_*i*_| denotes the number of connected edges between nodes in the neighborhood of node *i*, the number of triangles formed by node *i* and two nodes in its neighborhood, and *k*_*i*_ denotes the degree of node *i*. A complex network has a large number of nodes, so the study of the clustering coefficient of each node is more complex and meaningless. The definition of ACC is shown in Equation (10), which can measure the accumulation of the network. A high ACC represents a higher degree of a grouping of the whole network, and vice versa, a lower degree.

In a complex network, the edges a node needs to pass from one node to another are defined as paths, and the number of edges is defined as the path length. The path between nodes is not unique. There is a shortest path between two nodes, which is the shortest number of edges that need to be traversed. The average shortest path between all pairs of nodes in a network is defined as the CPL, which can be represented by Equation (11).


(11) $$\begin{array}{l} CPL=\frac{\underset{l\neq j}{\sum }L_{ij}}{N\left(N+1\right)} \end{array}$$

The *L*_*ij*_ represents the shortest path length from node *i* to node *j*. The CPL reflects the dispersion and connectivity of the network structure. The shorter the CPL in a network, the more stable the network and the higher the information transmission efficiency. The longer the CPL, the worse the network connectivity and the slower the communication speed between nodes.

MD is used to measure the possibility of a specific cluster in the network, which is called clustering strength in the network. The higher the MD, the clearer the community structure, and the smaller the mixing between communities. At this time, the proportion of edges within a community is more significant than between communities with the definition as Equation (12).


(12) $$\begin{array}{l} MD=\overset{M}{\underset{u=1}{\sum }}\left[e_{uu}-{\left(\overset{M}{\underset{v=1}{\sum }}e_{uv}\right)}^{2}\right] \end{array}$$

(13) $$\begin{array}{l} GE=\frac{1}{N\left(N+1\right)}\underset{i\neq j}{\sum }\frac{1}{L_{ij}} \end{array}$$

The network is divided into *M* modules. The *e*_*uv*_ refers to the proportion of the number of edges connected to the nodes in module *u* and the nodes in module *v* to the total number of edges. MD also defines the possibility that each node belongs to a community. Suppose there is a single isolated node in the network. In that case, the shortest path length from other nodes to this node tends to infinity, and the CPL is difficult to describe the network characteristics. Therefore, the GE must be introduced, as defined in Equation (13). It is the average value of the reciprocal of the shortest path length, which can be used to measure whether the network transmission and information processing process is efficient. High GE means that the network has high efficiency of information circulation and is more stable.

**Figure 6:**
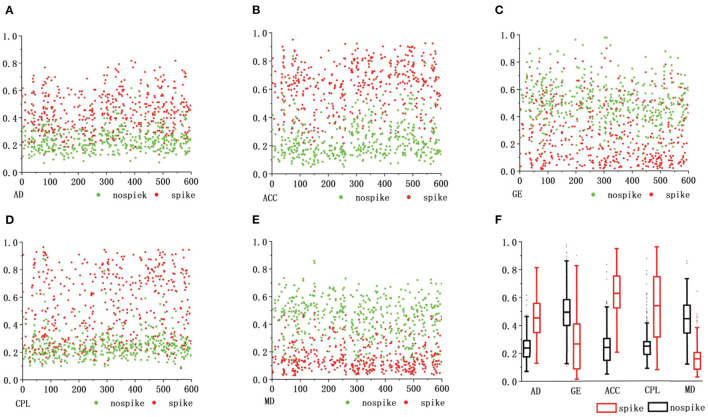
The abscissa of the comparison of functional brain network features. **(A–E)** Represents the recording time, the ordinate represents different functional brain network features, and the red dots represent the spike discharge and the corresponding FBN-PLV eigenvalues in this epoch. The green dots represent FPS discharge and the corresponding FBN-PLV eigenvalue in this epoch. The **(F)** is a comprehensive comparison of weighted network topology attributes.

Figure 6 shows the topological properties of the functional brain network corresponding to actual spike discharge and FPS discharge in the C3 channel and P3 channel in the first 600*s*, and carries on the normalization processing. It can be clearly seen that there is a big difference in the topological properties of FBN-PLV between the presence of spike discharge and the absence of spike discharge. Among them, the AD, ACC, and CPL of spike discharge are significantly increased. At this time, the brain information interaction mode is more complex, while the GE and MD show a downward trend.

### 2.5. ANN net-based classifier

#### 2.5.1. Network structure

ANN is a mathematical model that simulates the processing mechanism of the human brain nervous system to complex information after understanding and abstracting the response mechanism of the human brain structure to external stimuli on the basis of biology. It uses a hierarchical structure to construct a high-dimensional model (Lee and Lee, 2022). The model has parallel distributed processing capability, a simple structure, many network parameters, a large amount of calculation, and can withstand the scale of a multi-hidden layer network. Recently, it has been widely used in image recognition and audio processing.

**Figure 7:**
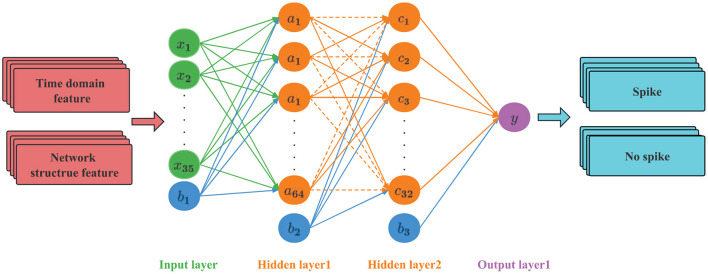
ANN network structure.

The ANN used in this article has a four-layer structure, as shown in Figure 7. The first layer is the input layer, the second and third layers are the hidden layers, and the fourth layer is the output layer. A Dropout layer is added to the hidden layer. The input layer sets 35 neurons to accept feature data. The hidden layer is the internal processing layer in the neural network. These neurons form an intermediate layer inside the network and are not directly associated with external input and output. Multiple hidden layers can abstract input features at multiple levels to linearly partition different types of data. In view of the moderate complexity of the epileptic EEG data set and the problems of overfitting and training difficulty caused by too many hidden layers, two hidden layers are set up, the first layer has 64 hidden nodes, and the second layer has 32 hidden nodes. The Dropout layer is mainly used to solve the overfitting problem. The principle is: using the regularization method, some neurons are randomly selected to output 0 in this iteration, but the weights are retained, and the nodes are reselected in the next iteration to participate in the weight update.

#### 2.5.2. Activation function

When training the neural network, the adjustment of hyperparameters is indispensable. The hyperparameters commonly used in deep learning neural networks are: gradient descent optimizer, loss function, and activation function. The activation function is often used to solve nonlinear problems, which determines the input of the next neuron and can increase the expression ability of the entire network. The common activation functions are Sigmoid, Relu, and Tanh. The definitions are shown in Equations (14–16). The Relu function is composed of two linear components. The output can be divided from 0 to infinity, and the convergence speed is faster than Sigmoid and Tanh. Therefore, the input and hidden layers use the Relu activation function. The classification of epileptic spikes belongs to the binary classification problem, so the output layer uses the Sigmoid activation function. The output is interpreted as a class label according to the probability value returned by the function.


(14) $$\begin{array}{l} Sigmiod\left(x\right)=\frac{1}{1+e^{-x}} \end{array}$$

(15) $$\begin{array}{l} Relu\left(x\right)=\{\begin{array}{cc} x, & \text{if}x>0\\ 0, & \text{if}x\leq 0 \end{array} \end{array}$$

(16) $$\begin{array}{l} Tanh\left(x\right)=\frac{e^{x}-e^{-x}}{e^{x}+e^{-x}} \end{array}$$

#### 2.5.3. Optimizer and learning rate

The definition of the loss function is the error between the single training sample and the actual value, which is used to evaluate the degree of deviation between the predicted value and the actual value of the model. The more appropriate the loss function is selected, the better the model's performance. The choice of loss function depends on many factors, which can be divided into two categories: classification loss and regression loss. The binary cross entropy loss function is commonly used in classification problems. It is defined as Equation (17), where *y* represents the label 0 or 1, and *p*(*y*) represents the probability that the output is 0 or 1. The greater the entropy of the probability distribution, the greater the uncertainty of the data distribution; similarly, the smaller the probability value, the more certain the distribution, the smaller the uncertainty.


(17) $$\begin{array}{l} Loss=-\frac{1}{N}\overset{N}{\underset{i=1}{\sum }}y_{i}\log\left(p\left(y_{i}\right)\right)+\left(1-y_{i}\right)\log\left(1-p\left(y_{i}\right)\right) \end{array}$$

The optimizer is a tool to guide the deep learning neural network to update the parameters. Using the optimization algorithm, starting from the loss value, the last layer is back-propagated to the front layer, and the weight and bias derivative of the network are calculated to complete the update of the network parameters. Adam is a first-order optimization algorithm that can replace the traditional stochastic gradient descent process. It can update the neural network weights in time based on training data iteration (Kingma and Ba, 2014). The traditional stochastic gradient descent maintains a single learning rate alpha and updates all weights. The learning rate does not change during the training process. However, Adam designs independent adaptive learning rates for different parameters by calculating the gradient's first-order moment estimation and second-order moment estimation. Assuming that the number of samples is *f*, the learning rate is η, β_1_ and β_2_ are two momentum hyperparameters that need to be adjusted, *g*_*new*_ and *v*_*new*_ are the modified first-order and second-order moments, and *w*_*new*_ represents the parameter update. The definition of the minimization objective function is shown in Equation (18):


(18) $$\begin{array}{l} Q\left(w\right)=\frac{1}{f}\overset{f}{\underset{i=1}{\sum }}Q_{i}\left(w\right) \end{array}$$

The Adma optimizer can be expressed as:


(19) $$\begin{array}{l} g_{new}=\frac{\beta_{1}g+\left(1-\beta_{1}\right)\nabla \hat{Q}\left(w\right)}{1-\beta_{1}} \end{array}$$

(20) $$\begin{array}{l} v_{new}=\frac{\beta_{2}g+\left(1-\beta_{2}\right)\nabla \hat{Q}{\left(w\right)}^{2}}{1-\beta_{2}} \end{array}$$

(21) $$\begin{array}{l} w_{new}=w-\eta \frac{g_{new}}{\sqrt{v_{new}+\varepsilon }} \end{array}$$

## 3. Results

### 3.1. Subjects and data acquisition

The EEG signals used in this study are recorded by digital EEG instruments in the Children's Hospital, Zhejiang University School of Medicine. A total of 4 patients are children, but the patient's names and age information are hidden. The EEG acquisition method is 10–20 international standard lead system, including 21 electrodes, including A1 and A2 reference electrodes, and the sampling rate is 1,000*Hz*. Due to the baseline effect of the device itself, as well as factors such as muscle activity and blinking, noise and artifacts are generated in EEG signals. Therefore, the Butterworth filter is used to filter the EEG signals of 0.017–70 *Hz* (Wang et al., 2020). The pathogenesis of epileptic spines usually originates from a part of the brain, including the frontal, central, parietal and temporal regions. The EEG signals of 4 patients with BECT are analyzed. It is found that spike discharges of these EEG signals had specific attack areas. The four EEG data used in this study are named S1-S4. Table 2 shows the recorded scalp EEG information of 4 patients, including recording duration, the number of spikes, and spike position. Each EEG signal contains an average AV channel (FP1, FP2, F3, F4, F7, F8, T3, T4, T5, T6, C3, C4, P3, P4, O1, and O2), and professional electroencephalogram technician marked spike discharge position.

**Table 2:** Comparison of raw data of spikes (actual peaks) and candidate spikes obtained by specific template matching (candidate peaks).

**Name**	**Duration of data**	**Actual peaks**	**Candidate peaks**	**Regions**
S1	20 min 20 s	C3(147), P3(134)	C3(2152), P3(2477)	C3, P3
S2	20 min 12 s	C3(327), P3(277)	C3(2395), P3(2445)	C3, P3
S3	20 min 12 s	C3(197), P3(203)	C3(1582), P3(1491)	C3, P3
S4	19 min 56 s	C3(437), P3(437)	C3(1101), P3(1197)	C3, P3

The candidate spike set obtained by preliminary screening through specific template matching is also shown in Table 2. There are a large number of spike signals in the candidate spike set, of which only a few are spikes and a large number are FPS. Such data sets need to be more balanced. Therefore, we use the random undersampling method for FPS data to obtain a positive and negative sample balanced candidate spike data set *S*_*all*_.

**Figure 8:**
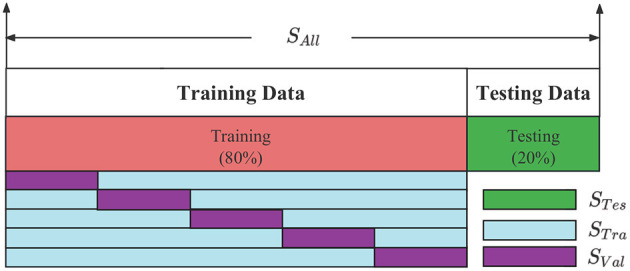
The *S*_*Tra*_, *S*_*Val*_, *S*_*Tes*_ division of BECT data.

After obtaining a balanced positive and negative sample *S*_*All*_, it is segmented twice. The *S*_*All*_ divides the data set into a training set and a test set, where 80% of the data is used for training and 20% for testing, as shown in Figure 8. The *S*_*Tes*_ represents the test set, which is used to evaluate the model after training and test the final performance of the model. The *S*_*Tra*_ represents the training set to train the model and update the model parameters. The *S*_*Val*_ represents the validation set, which is used to observe the training effect of the model. The *S*_*Tra*_ and *S*_*Val*_ are set according to the 5-fold cross-validation method. The model structure and hyperparameters are adjusted according to the loss value and accuracy.

### 3.2. Evaluation criteria

The quality of the model requires an objective evaluation standard. For the classification model, the confusion matrix can intuitively calculate the accuracy (AC), specificity (SP), sensitivity (SE), and other indicators of the model. Equation (22), where TN is the number of correctly detected FPS, FN is the number of missed spikes, TP is the number of correctly detected spikes, and FP is the number of incorrectly detected spikes. As shown in Equation (23), the AC reflects the percentage of samples that predict correctly. SE refers to the ratio of the number of detected correct spikes to the number of all spikes, which is the proportion of the number of actual spikes detected, as defined in Equation (24). SP refers to the ratio of the number of correctly detected FPS to the number of all FPS, as defined in Equation (25). SE and SP represent the detection ability of the positive and negative samples of the model, respectively.


(22) $$\begin{array}{l} CM=\left[\begin{array}{cc} TN & FP\\ FN & TP \end{array}\right] \end{array}$$

(23) $$\begin{array}{l} AC=\frac{TN+TP}{TN+FN+TP+FP}\times 100\% \end{array}$$

(24) $$\begin{array}{l} SE=\frac{TP}{FN+TP}\times 100\% \end{array}$$

(25) $$\begin{array}{l} SP=\frac{TN}{TN+FP}\times 100\% \end{array}$$

### 3.3. Experimental results

**Figure 9:**
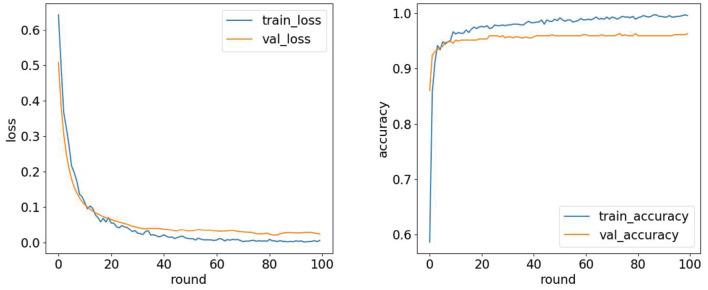
Loss value and accuracy under different iterations.

The experimental computer configuration is as follows: 11th Gen Intel (R) Core (TM) i5-11400H @ 2.70GHz 2.69GHz, 16GRAM; the simulation environment is python3.7. A round represents training once with all the samples in the training set, and the value of round is the number of times the entire data set is trained. We use different rounds on the training and validation sets to observe the changes in loss values and accuracy, as shown in Figure 9, and then select the most appropriate number of rounds. The validation and training sets' loss values decreased rapidly in the first 20 rounds. When the number of rounds is 100, the model performance tends to be stable, and the number of rounds is finally selected to be 100.

**Figure 10:**
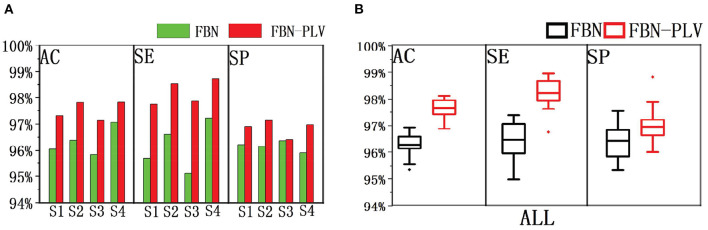
The performance comparison between FBN and FBN-PLV models. **(A)** AC, SE, and SP of the actual EEG data of each patient after using the method of this paper. **(B)** AC, SE, SP after synthesis of actual EEG data for all patients.

Based on the data set division method mentioned above, after selecting the best parameters of the model, divide different training sets and test sets each time, and run 100 times in total. The experimental results are shown in Figure 10. FBN refers to the model's performance on the EEG data of each patient when there are only time-domain characteristics. FBN-PLV represents the model's performance on EEG data of each patient when inputting time-domain features and functional network features. The overall AC, SE, and SP are calculated by adding the confusion matrix of each patient. The input of functional network features and time-domain features significantly improves the performance of the whole model compared with the input of only time-domain features. The average results of these 100 experiments are shown in Table 3. The addition of PLV features improved the model's accuracy by 1.33%, sensitivity by 1.70%, and specificity by 0.63%. In auxiliary medical detection and other related fields, SE means whether the model can effectively detect positive cases. The significant improvement of SE also reflects the significance of the characteristics of the functional brain network.

**Table 3:** Model performance comparison between FBN and FBN-PLV.

**Feature**	**Patients**	**FN**	**AC (*%*)**	**SE (*%*)**	**SP (*%*)**
FBN	S1	18	96.05	95.69	96.21
	S2	7	96.37	96.60	96.15
	S3	19	95.82	95.11	96.35
	S4	9	97.07	97.21	95.92
	ALL	53	96.27	96.54	96.20
FBN-PLV	S1	10	97.32	97.75	96.90
	S2	3	97.83	98.53	97.14
	S3	7	97.13	97.88	96.40
	S4	4	97.84	98.74	96.97
	ALL	24	97.60	98.24	96.83

**Figure 11:**
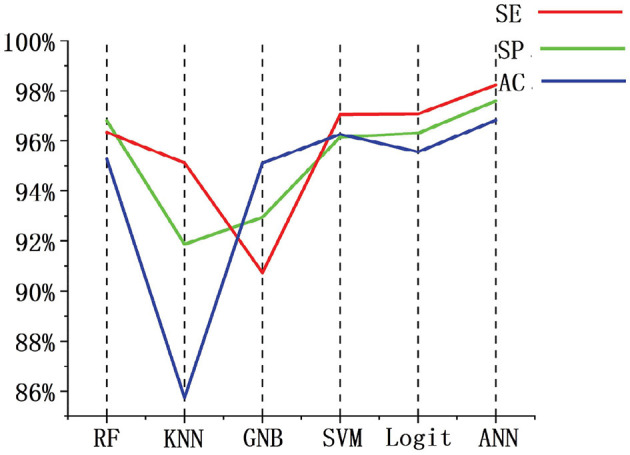
Performance comparison of different classifiers based on FBN-PLV.

We used the same data set partitioning method and data set as in this paper. The performance of random forest (RF), K-Nearest neighbor (KNN), Gaussian naive bayes (GNB), SVM, and Logit were compared with the indexes of the algorithms presented in this paper. Figure 11 shows the performance comparison of FBN-PLV features on different classifiers and then observes the evaluation index of each classifier. The specificity of the RF classifier is slightly higher than ANN, but the AC and SE are lower. In general, the ANN classifier based on FBN-PLV has achieved the best results on the actual EEG data of BECT.

## 4. Discussion

The algorithm is compared with the related algorithms of other researchers, and the results are shown in Table 4. In addition to Chahid et al.'s position weight matrix (PWM) method, other methods are EEG-based (Chahid et al., 2020). Our proposed FBN-PLV method has better AC, SE, and SP than all EEG-based methods. Due to the spikes' relatively clear morphological definition, the candidate spike set is obtained by triangle-specific matching and threshold selection. The time-domain feature extraction of spike morphology is a more efficient spike screening method. At the same time, because of the temporal and spatial correlation between brain regions, especially the dispersion of spikes showing a “peak-to-peak” mode, the spikes show a synchronous characteristic in phase. Accordingly, this paper proposes the construction method of FBN-PLV, using PLV to calculate the synchronization characteristics between channels and establish phase synchronization FBN. The AD, ACC, CPL, MD, and GE are calculated according to the established network. As shown in Figures 10, 11, the functional brain network characteristics improve the model's performance. In addition, the ANN is also the most suitable model for the algorithm in this paper. The final results obtained in the data set test are AC: 97.60%, SE: 98.24%, and SP: 96.83%, which is the best among the listed spike recognition methods based on EEG data.

**Table 4:** Performance comparison of recent automatic spike detection methods.

**References**	**Feature**	**Classifier**	**Number of patients**	**AC(*%*)**	**SE(*%*)**	**SP(*%*)**
Prasanth et al. (2020)	Frequency sub-bands	CNN	–	–	90.00	–
Xu et al. (2021)	Time domain, SEN	Bi-LSTM	15	92.04	92.04	85.05
Wang et al. (2020)	Adaptive template matching, FPS elimination	RF	7	96.90	97.40	96.50
Dao et al. (2019)	Fisher score, p-value	SVM	6	93.80	82.80	96.40
Le et al. (2018)	Time domain	DBN 16	–	87.40	97.90	
Thanh et al. (2020)	Eigenspikes derived from nonnegative GSMLRAT	–	17	91.00	83.00	91.10
Rácz et al. (2020)	Features from RNN and CNN	CNN	–	89.00	69.00	–
Chahid et al. (2020)	PWM	SVM	8	**98.22**	98.06	**98.38**
Cheng et al. (2022)	Multilevel learning features	–	8	90.62	90.38	91.00
**Our method**	Time domain, network structrue	ANN	4	97.60	**98.24**	96.83

The bold values represent the best results.

It is worth noting that reference (Chahid et al., 2020) proposed an automatic detection algorithm for epileptic spikes based on machine learning magnetoencephalography (MEG), using PWM method combined with a uniform quantizer to generate useful features and using SVM for classification. The literature has achieved higher AC and SP than the algorithm in this paper. However, compared with EEG, MEG is affected by physical activity, and it is difficult to locate epileptic foci and functional areas accurately. The process of extracting MEG requires high cooperation from patients. Finally, the SE of this paper is higher than that of the literature (Chahid et al., 2020). In auxiliary medical detection, the importance of SE is much greater than SP and AC. The algorithm achieves the best SE of 98.3% in all algorithms and also ensures high SP and AC.

## 5. Conclusion

The main contributions of this paper are as follows: (1) A phase synchronization functional brain network is proposed, and the weighted network structure features are extracted to study the attribute changes caused by BECT spike discharge from this perspective. (2) An artificial neural network deep learning model is used to detect spikes intelligently by combining time domain features with phase synchronization functional brain network features. The proposed method is tested on the actual patient EEG data set provided by the Children's Hospital, Zhejiang University School of Medicine, and achieves superior performance, in which the SE is significantly improved. This paper only validates the algorithm on EEG datasets from four BECT patients, and each dataset contains only a limited number of spike discharge samples. While good results have been achieved, further optimization and improvement are needed to enhance the versatility and robustness of the spike detection algorithm. The FBN-PLV can enhance the accuracy of spike detection to some extent, but it only detects the presence of spike discharge, which is insufficient to pinpoint the specific location of spike discharge. In the future, more ways can be used to construct functional brain networks, study the differences before and after epileptic spike discharges, and optimize algorithms for more types of EEG data except for BECT data sets.

## Data availability statement

The original contributions presented in the study are included in the article/supplementary material, further inquiries can be directed to the corresponding author.

## Ethics statement

This research has been approved by Ethics Committee of the Children's Hospital, Zhejiang University School of Medicine (2019-IRB-152).

## Author contributions

LJ, QF, and JL contributed to conception and design of the study. JR analyzed the data. LJ and QF wrote the first draft of the manuscript. FD improved sections of the manuscript. TJ provided data set and medical advice. All authors contributed to manuscript revision, read, and approved the submitted version.
